# Targeted mutagenesis in chicken using CRISPR/Cas9 system

**DOI:** 10.1038/srep23980

**Published:** 2016-04-06

**Authors:** Isao Oishi, Kyoko Yoshii, Daichi Miyahara, Hiroshi Kagami, Takahiro Tagami

**Affiliations:** 1 Biomedical Research Institute, National Institute of Advanced Industrial Science and Technology, 1-8-31, Midorioka, Ikeda, Osaka 563-8577, Japan; 2 Faculty of Agriculture, Shinshu University, 8304 Minamiminowa, Nagano 399-4598, Japan; 3 Animal Breeding and Reproduction Research Division, National Agriculture and Food Research Organization, Institute of Livestock and Grassland Science, 2 Ikenodai, Tsukuba, Ibaraki 305-0901, Japan

## Abstract

The CRISPR/Cas9 system is a simple and powerful tool for genome editing in various organisms including livestock animals. However, the system has not been applied to poultry because of the difficulty in accessing their zygotes. Here we report the implementation of CRISPR/Cas9-mediated gene targeting in chickens. Two egg white genes, *ovalbumin* and *ovomucoid*, were efficiently (>90%) mutagenized in cultured chicken primordial germ cells (PGCs) by transfection of circular plasmids encoding Cas9, a single guide RNA, and a gene encoding drug resistance, followed by transient antibiotic selection. We transplanted CRISPR-induced mutant-*ovomucoid* PGCs into recipient chicken embryos and established three germline chimeric roosters (G0). All of the roosters had donor-derived mutant-*ovomucoid* spermatozoa, and the two with a high transmission rate of donor-derived gametes produced heterozygous mutant *ovomucoid* chickens as about half of their donor-derived offspring in the next generation (G1). Furthermore, we generated *ovomucoid* homozygous mutant offspring (G2) by crossing the G1 mutant chickens. Taken together, these results demonstrate that the CRISPR/Cas9 system is a simple and effective gene-targeting method in chickens.

Chicken is a commercially important animal and its genetic modification is expected to be used for agricultural, industrial, and scientific applications^1^,^2^,^3^. There are various possible and beneficial applications of genetically modified chicken, including improvement of production of meat and eggs, generation of disease-resistant chickens, mass production of therapeutic proteins in egg whites, and establishment of models for studying avian development. Furthermore, gene disruption of egg white allergen genes such as *ovalbumin* (*OVA*) and *ovomucoid* (*OVM*) has the potential to produce low allergenicity in eggs, thereby reducing immune responses in individuals sensitive to items such as egg white–containing food products and vaccines. To produce these genetically modified chickens, efficient technologies, including suitable transgenic and knockout methods, are required. However, the genetic modification of chickens has lagged far behind that of other organisms because of the difficulty in accessing and manipulating the zygote^4^. Therefore, chicken transgenesis has mainly been performed using viral vector infection of the early stage embryo^5^,^6^. Recently, primordial germ cells (PGCs), which can be cultured and genetically modified *in vitro*, have been used to generate transgenic chickens by taking advantage of their germline competency after injection into recipient embryos^7^,^8^,^9^,^10^,^11^. In contrast to dozens of studies generating transgenic chickens with these methods, few reports are available on chicken gene disruption. The generation of knockout chickens was first reported by Schusser *et al.*^12^. In this study, the immunoglobulin heavy chain gene was disrupted in cultured PGCs by homologous recombination with a targeting construct. Thereafter, heterozygous and homozygous gene-targeted chickens were produced in G1 and G2 offspring of germline chimeric chickens that had been transplanted with the gene-disrupted PGCs. Recently, Park *et al.* reported *OVA* heterozygous knockout chicken produced by transcription activator-like effector nuclease (TALEN)-induced PGC mutation^13^. This study was the first to demonstrate the effectiveness of site-specific nuclease-mediated genome-editing technology in the generation of mutant chickens.

Another genome-editing technology is the clustered regularly interspaced short palindromic repeats (CRISPR)-associated protein system, known as CRISPR/Cas^14^,^15^. CRISPR/Cas9 uses an RNA-guided nuclease (Cas9) to target specific sequences and induces DNA double-stranded breaks (DSBs) therein. During the DSB repair process by non-homologous end-joining (NHEJ), small insertions or deletions (indels) are efficiently introduced, and the indels can lead to shifts in the reading frame and the ultimate functional disruption of targeted proteins. Because the CRISPR/Cas9 system requires only a pair of oligonucleotides containing the target sequence, preparation of the plasmid for targeted gene disruption is much easier and more cost-effective compared with the TALEN-mediated method^14^,^15^. To date, various organisms have been mutagenized using the CRISPR/Cas9 system^16^, including livestock animals such as pig^17^, rabbit^18^, and goat^19^, but not avian species. Therefore, the methods should be developed to apply the CRISPR/Cas9 system to generate gene targeting in chickens.

Here we report successful gene targeting of *OVM* in the chicken using the CRISPR/Cas9 system. A single plasmid transfection followed by antibiotic selection resulted in targeted mutation with >90% efficiency in chicken PGCs. The mutated PGCs generated functional gametes via germline chimera and produced male and female G1 offspring with various *OVM* mutations. In addition, OVM^−/−^ mutant chickens were obtained as G2 offspring by crossing OVM^+/−^ mutant chickens.

## Results

### Validation of single guide RNA (sgRNA) disruption of *OVA* and *OVM*

**Figure 1:**
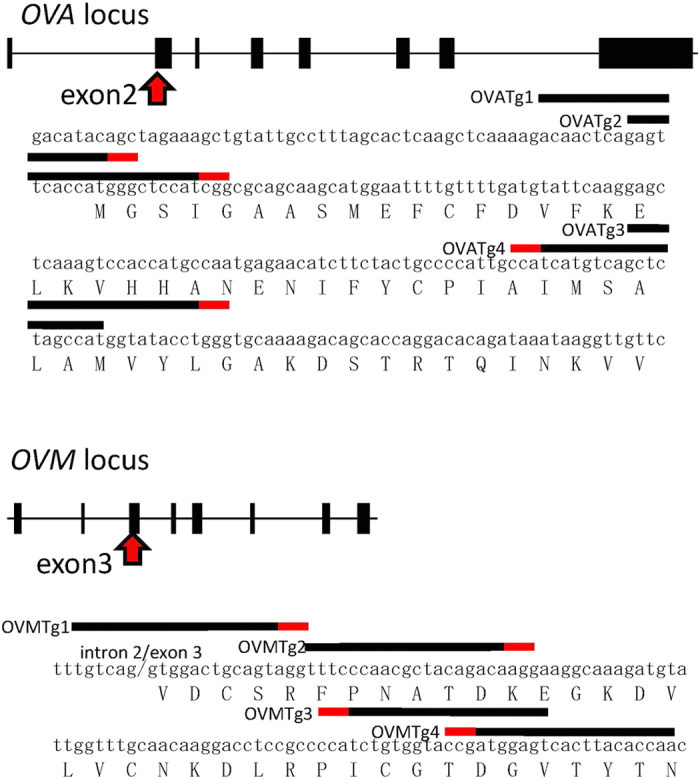
Schematic representation of sgRNAs targeting the *OVA* and *OVM* loci. Exon-intron organization and targeting sequences for the *OVA* (upper) and *OVM* (lower) loci are shown. DNA and amino acid sequences are shown in lowercase and uppercase letters, respectively, and correspond to the regions indicated by the red arrows. The four sgRNA targeting sites are numbered and represented by black bars above the nucleotide sequence. Adjoining protospacer adjacent motif (PAM) sequences are highlighted in red. The OVMTg1 target site spans the intron 2/exon 3 boundary.

**Figure 2:**
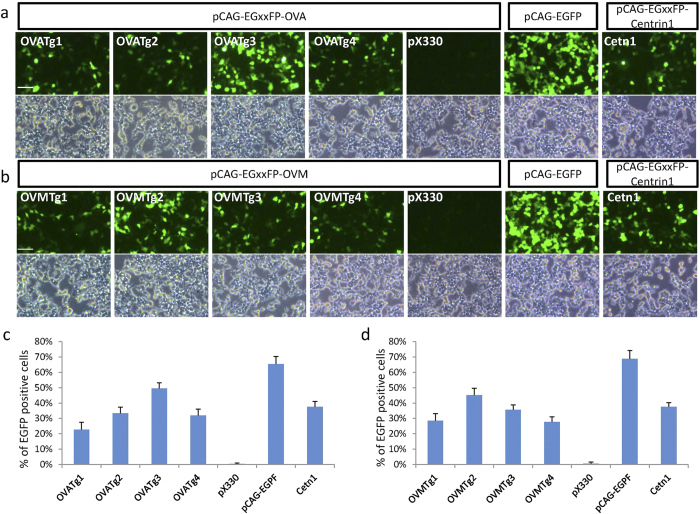
Validation of sgRNA target sites for disruption of chicken *OVA* and *OVM* by SSA recombination assay. (**a,b**) Fluorescence (top) and phase contrast (bottom) microscopy images of HEK293T cells transfected with pCAG-EGxxFP reporters and pX330 plasmids at 48 h post-transfection. (**a**) Co-transfection with pCAG-EGxxFP-OVA and pX330 plasmids containing *OVA* sgRNA sequences OVATg1–4 or pX330 without sgRNA sequences. (**b**) Co-transfection with pCAG-EGxxFP-OVM and pX330 plasmids containing *OVM* sgRNA sequences OVMTg1–4 or pX330 without sgRNA sequences. Right two images in (**a,b**) show EGFP fluorescence generated by co-transfection with pCAG-EGxxFP-Centrin1 (Cetn1) and pX330/Cetn1 plasmids as an experimental control and by transfection with pCAG-EGFP as a positive control. Scale bar = 100 μm. (**c,d**) Percentage of EGFP-positive cells relative to all cells from experiments carried out as in (**a,b**), which reflects the efficiency of DSB-mediated homology-dependent repair. Data are shown as the mean ± standard deviation (SD) from four regions of a single culture.

To use the CRISPR/Cas9 system for targeted mutagenesis in chickens, which will lead to the improvement of chicken products, we chose two egg white genes, *ovalbumin* (*OVA*) and *ovomucoid* (*OVM*), as targets for disruption. Four targeting (Tg) sites close to the initiation codon were selected for each gene: OVATg1–4 in exon 2 of *OVA,* and OVMTg1–4 in exon 3 of *OVM*, and sgRNAs were designed for each target sequence (Fig. 1). Eight neomycin resistance–based plasmids encoding Cas9 and the specific sgRNAs (pX330-Neo-OVATg1–4 and pX330-Neo-OVMTg1–4) were generated and tested for their ability to disrupt the target gene by the single-strand annealing (SSA) assay^20^. The reporter plasmid pCAG-EGxxFP-OVA or pCAG-EGxxFP-OVM, which contains the target region for *OVA* or *OVM*, respectively, was co-transfected with each pX330 plasmid into HEK293T cells. Two days after transfection, cells were assayed for DSB repair of target genes using reconstituted EGFP fluorescence as the readout. The results are shown in Fig. 2a,b. The average fluorescence intensity was evaluated for each plasmid, and from these results two candidates, the OVATg3 and OVMTg2 versions of the pX330-Neo plasmids were selected as most efficient in disrupting *OVA* and *OVM*, respectively (Fig. 2c,d).

### *OVA* and *OVM* mutation by CRISPR/Cas9 in chicken PGCs

**Figure 3:**
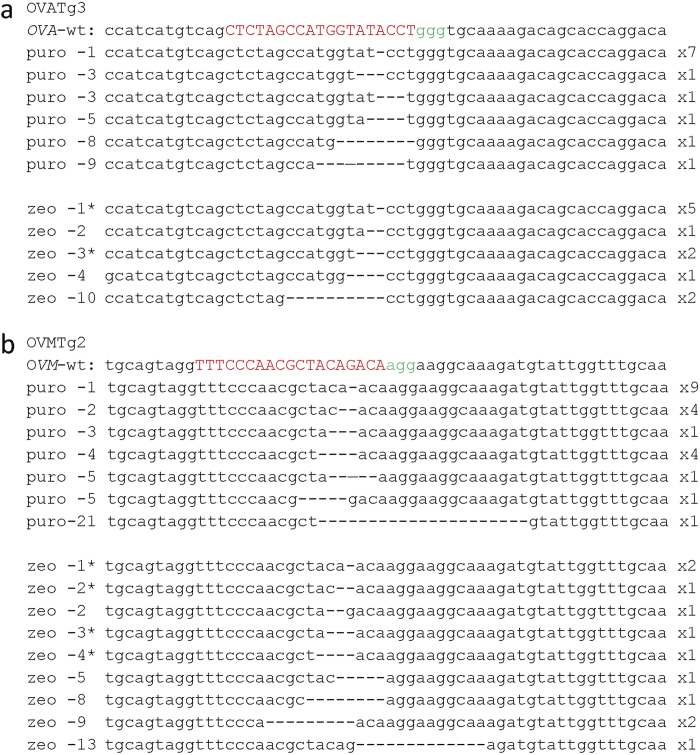
CRISPR/Cas9-mediated mutation of *OVM* and *OVA* in PGCs. (**a,b**) Sequence analysis of OVATg3-induced (**a**) and OVMTg2-induced (**b**) deletion mutations in PGCs. The wild-type sequence (OVA- and OVM-WT) is shown at the top of each panel. The sgRNA-targeted locus is indicated in capital letters in red; the PAM sequence is in green. Antibiotics used for PGC selection are indicated as puro (puromycin) and zeo (zeocin). The number of deleted nucleotides (−1 to −21) is indicated to the left of each sequence. An asterisk indicates that the same deletion mutant was observed in puromycin-selected PGCs. Deleted nucleotides are shown by dashes. The number of identical mutant clones is shown to the right of each sequence.

We next examined whether *OVA* and *OVM* in chicken PGCs could be disrupted by transfection with the two selected pX330 plasmids. Cultured PGCs derived from Barred Plymouth Rock (BPR) male embryos were transiently transfected with pX330-Neo-OVATg3 or pX330-Neo-OVMTg2, selected with or without 0.5 mg/ml neomycin at 2–4 days post-transfection, and then cultured for up to 2 weeks without neomycin. A subset of the PGCs was collected, and *OVA* and *OVM* sequences around target sites were PCR amplified and analyzed following TA cloning. We found *OVA* and *OVM* mutations with frequencies of 34% and 13%, respectively, in the TA clones derived from neomycin-treated PGCs in contrast to respective mutation frequencies of 6.7% and 0% without neomycin selection (Table 1). Given that neomycin treatment resulted in more-efficient mutation of the two genes, transient antibiotic selection was efficacious for CRISPR/Cas9-mediated gene editing in PGCs. To potentially increase the proportion of mutant alleles in PGCs with the CRISPR/Cas9 method, we tested two additional antibiotic selection techniques, puromycin and zeocin, after insertion of their corresponding resistant genes into the pX330 plasmid. As shown in Table 1, transient antibiotic selection at 2–4 days post-transfection with puromycin (1 μg/ml) and zeocin (50 μg/ml) resulted in mutational frequencies of 100% and 92% in *OVA* and 91% and 92% in *OVM*, respectively. These high frequencies of mutations with puromycin and zeocin as compared with neomycin can be attributed to the differences in antibiotic sensitivities of untransfected PGCs. Cultured chicken PGCs were rather more sensitive to puromycin and zeocin than to neomycin (Fig. S1). Thus PGCs in which the pX330 plasmid was not successfully transfected might survive after the transient treatment of neomycin as compared with exposure to puromycin and zeocin. In the sequenced clones, 9 mutational classes with 1- to 10-bp deletions in *OVA* and 12 classes with 1- to 21-bp deletions in *OVM* were observed (Fig. 3). Notably, although various deletion mutations were detected, no insertion mutations were found in the analyzed sequences. The majority of these deletions induced frameshift mutations and consequent changes in OVA and OVM proteins. These results show that endogenous genes can be efficiently targeted by CRISPR/Cas9 mutagenesis by using proper antibiotic selection in chicken PGCs. Consistent with this idea, expression of a PGC-specific endogenous protein, chicken vasa homolog (CVH)^21^, was frequently reduced or absent after transfection of a pX330-Puro plasmid encoding CVH-specific sgRNAs followed by transient selection with puromycin (Fig. S2).

### Generation of *OVM* heterozygous mutant chickens

**Figure 4:**
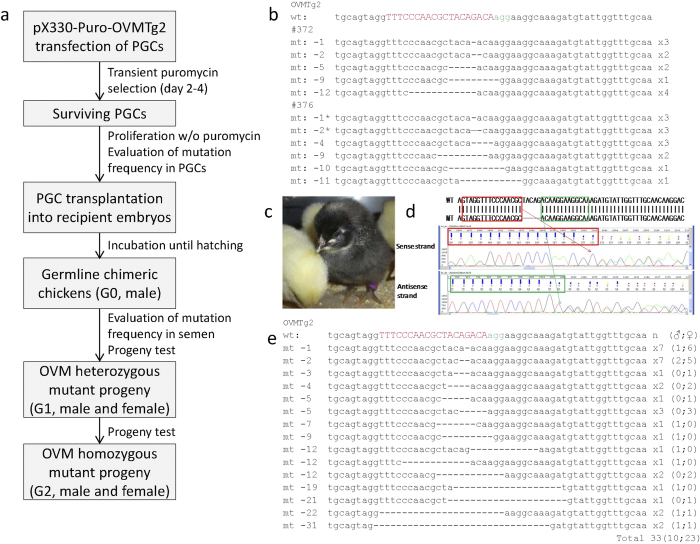
CRISPR/Cas9-mediated mutation of *OVM* in G0 semen and donor PGC–derived G1 offspring. (**a**) Schematic representation of the experimental process to generate *OVM* mutant chickens with CRISPR/Cas9. (**b**) *OVM* mutations in semen of chimeric chickens. Top: wild-type (wt) *OVM* sequence is shown in black with the sgRNA-targeted locus shown in uppercase red lettering, the PAM sequence in green, and the deleted nucleotides as dashes. Nine types of mutation in semen from two chimeric chickens, #372 and #376, are indicated. The number of deleted nucleotides (−1 to −12) is indicated to the left of the sequence. The number of mutant clones obtained is shown to the right of each sequence. Numbers followed by an asterisk in the #376-derived mutants refer to the identical deletion in the #372-derived mutants. (**c**) Chicks that hatched from a test-cross from a male germline chimera. Black feather color (i/i) identifies chicks derived from donor-BPR PGCs, whereas white feather color (I/i) marks offspring derived from recipient-WL PGCs. (**d**) *OVM* genomic sequence from the black chick shown in (**c**). The heterozygous 5-bp deletion was detected by sense and antisense sequencing. Red and green boxes in the sequence alignment (top) refer to the corresponding sequences in the sense and antisense sequence analysis (bottom). Red and green arrows indicate the 3′ and 5′ ends, respectively, of the 5-bp deletion observed in this chick genome. (**e**) Sequences of *OVM* mutations observed in PGC-derived offspring. Top: wild-type sequence is shown at the top and isolated mutants are shown below. Sequences were annotated as in (**b**), and the number of chickens with identical mutations is shown to the right of each sequence (males and females).

Cultured chicken PGCs differentiate into functional gametes after transplantation into recipient chicken embryos^7^. To determine whether the PGCs carrying CRISPR/Cas9-induced mutations have germline competency and the capacity to generate offspring, we produced germline chimeric chickens and analyzed their offspring (Fig. 4a). Because *OVA* mutant chickens had already been generated^13^, we focused on the generation of novel *OVM* mutants. PGCs from the same pool of cells that were transfected with pX330-Puro-OVMTg2, transiently selected with puromycin, and sequenced were used as donor cells, and thus the vast majority (~90%) were expected to contain mutated *OVM*. The donor PGCs, which were derived from the BPR strain and thus carry the recessive gene (i/i) for black feather color, were expanded and transplanted into the blood of recipient chick embryos from the White Leghorn (WL), which carries the gene for white feather color (I/I). Prior to transplantation, recipient WL embryos were exposed to 5–6 Gy gamma radiation to ablate endogenous PGCs and increase the contribution from donor PGCs^8^. Three presumptive male germline chimeras (G0) were raised to sexual maturity, and the *OVM* genomic DNA in their semen was PCR amplified, TA cloned, and sequence analyzed. More than 90% of TA clones from two roosters (#372 and #376) exhibited *OVM* mutations with 1- to 12-bp deletions around the OVMTg2 target site (Table 2 and Fig. 4b). These two roosters were crossed with wild-type BPR hens (i/i), and the chicks that hatched were analyzed for feather color. Analysis of the progeny from the roosters demonstrated that the frequency of donor-derived (i/i) progeny as assayed by black feather color was 79% (#372) and 67% (#376). Sequence analysis revealed that 58% (#372) and 48% (#376), respectively, of the donor-derived progeny had an *OVM* mutation with a 1- to 31-bp deletion (Table 2 and Fig. 4c–d). These results indicate that the donor PGCs mutagenized by the CRISPR/Cas9 system could differentiate into functional gametes and produce genetically modified progeny. From among the monoallelic *OVM* mutant chicks, males and females with frameshift mutations were selected as heterozygous *OVM* knockout (*OVM*^+/−^) chicks. All the *OVM*^+/−^ chicks were healthy and showed no obvious abnormalities.

### Off-target and plasmid integration analysis

In previous studies, frequent off-target mutations have been observed in CRISPR-mediated mutagenesis in mammalian cell lines^22^,^23^. To evaluate the off-target effects of CRISPR in chickens, we analyzed potential off-target sequences in the genomes from these mutant chickens. Three candidates were chosen, as they carried sequences that matched the OVMTg2 seed sequence: two covered a 12-nucleotide stretch followed by the 5′-NGG-3′ protospacer adjacent motif (PAM) sequence; the other, a span of 17 nucleotides, was followed by the 5′-NAG-3′ PAM sequence (Table S2). These sequences were PCR amplified and analyzed by sequencing. For a total of 43 sequences from three potential off-target sites present in 19 mutant chickens, we did not detect any mutations (Table S2). These off-target mutations were not observed in genomic DNA from transfected PGCs that had not been transplanted into recipient embryos (Table S2). These results suggest that off-target mutations occur infrequently in the *OVM* mutant chickens and PGCs generated by the CRISPR/Cas9 system. To analyze the possibility of plasmid integration in knockout chicks, we searched for the puromycin resistance gene in the genome of *OVM*^+/−^ chicks by PCR analysis (Fig. S3). As the designed primer set could detect 10 fg of plasmid DNA, this assay should be able to detect as few as 0.05 copies of the puromycin resistance gene when 10 ng of genomic DNA is analyzed. No obvious transgene product was detected in eight *OVM*^+/−^ chicks, suggesting that random plasmid integration in the G1 mutated chicken genome occurs at a low frequency. We also examined puromycin sensitivity of PGCs that were transfected but not subsequently transplanted (Fig. S3). The PGCs were sensitive to puromycin, suggesting that random integration of the plasmid rarely occurred in the PGCs.

### Generation of *OVM* homozygous mutant chickens

**Figure 5:**
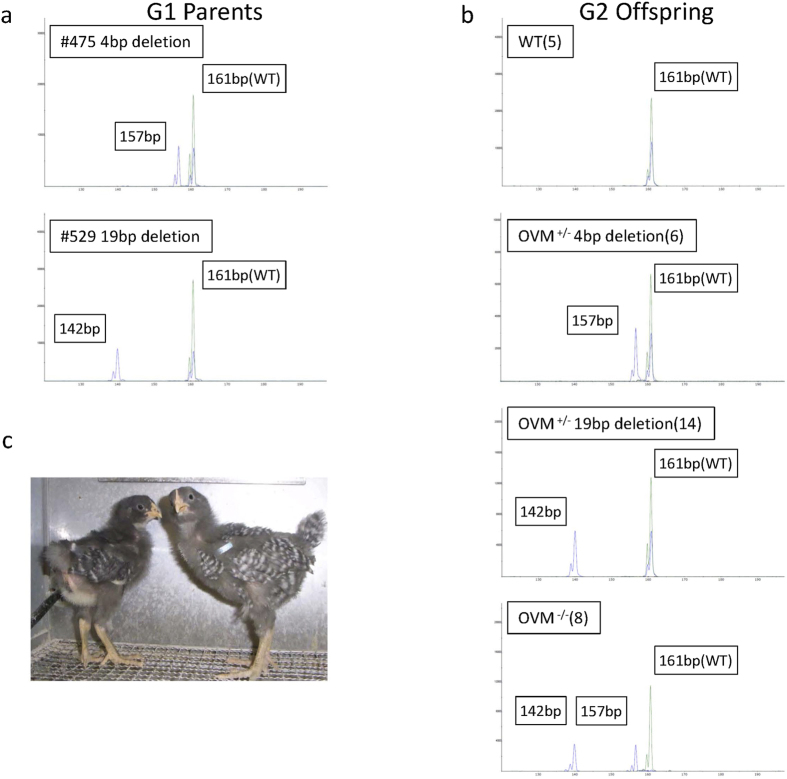
CRISPR/Cas9-mediated mutation of *OVM* in G2 offspring. (**a,b**) Gene fragment analysis for *OVM* in G1 heterozygous mutant parents (4-bp and 19-bp deletions) and their offspring (G2) are shown. Wild type and heterozygous and homozygous mutants were detected among the G2 animals; the number of identical G2 offspring is shown in parentheses next to each label. The *x* axis indicates amplicon size in base pairs, and the *y* axis indicates relative fluorescence intensity of PCR signal to loading control. The signal from the 6-FAM–labeled amplicon from the sample and HEX-labeled wild-type amplicon are indicated by a blue and green line, respectively. The size of each amplicon (in base pairs) is shown. (**c**) OVM^−/−^ male (right) and female (left) chick (23 days old after hatching).

Finally, we examined whether homozygous *OVM* knockout chickens can be generated by crossing *OVM*^+/−^ males and females. A sexually mature G1 male (#529) and G1 female (#475), which have an *OVM* mutation with a 19- and 4-bp deletion, respectively, were crossed, and the genomes of G1 parents and G2 progeny were analyzed by fragment analysis^24^ (Fig. 5a,b). As shown in Fig. 5b, the frequency of *OVM*^−/−^ progeny was 24% (8/33), corresponding to Mendelian inheritance. Both male and female *OVM*^−/−^ chicks hatched and were healthy (Fig. 5c). This result indicated that the G1 mutations introduced with the CRISPR/Cas9 system do not noticeably affect chromosomal distribution during meiosis or gamete differentiation.

## Discussion

To our knowledge, this study represents the first example of the generation of CRISPR/Cas9-mediated mutant chickens. A single plasmid transfection and transient antibiotic selection resulted in efficient targeted mutation in PGCs (>90%), and, therefore, chimerism of spermatozoa in PGC-transplanted G0 chimera chickens could easily be evaluated. The percentage of heterozygous *OVM* mutant chicks among the donor-derived offspring (G1) was around 50% from two of the three G0 founders (Table 2), and the majority of mutants resulted in frameshift mutations (Fig. 4e). In addition, *OVM*^−/−^ mutant offspring (G2) were obtained by crossing a *OVM*^+/−^ male and female G1 chicken, indicating that the CRISPR/Cas9 system is quite useful for generating gene-specific knockout in chickens. To date, conventional homologous recombination–based approaches and the TALEN method have been used to generate knockout chicken^12^,^13^. Because CRISPR/Cas9-mediated knockout requires simple plasmid construction compared with the other methods^14^,^15^, our results validate this as a convenient and easy-to-use method for gene targeting in chickens.

In contrast to several other organisms, zygote manipulation in chickens has rarely been successful^4^. Therefore, instead of zygotes, germline-competent cultured PGCs have been used to generate genetically modified chickens. Several transgenic and knockout chickens were generated by gene alteration of PGCs followed by transplantation of the PGCs into recipients, rearing of the recipients to sexual maturity, and identification of the genetically modified offspring^7^,^8^,^9^,^10^,^11^,^12^,^13^. Here we showed induction of PGC mutations in two genes with >90% efficiency using the CRISPR/Cas9 system in combination with antibiotic selection (Table 1). Park *et al.* have investigated PGC mutation using TALEN with fluorescence-activated cell sorting selection and established mutant offspring. They observed PGC mutation in *OVA* with 33.3% efficiency^13^. The differences in the mutational frequencies between the two studies reflect the differential mutational efficiencies of TALEN and CRISPR/Cas9. Consistent with this idea, comparative analyses of TALEN- and CRISPR/Cas9-mediated genetic mutation have shown a superior mutational frequency induced by CRISPR/Cas9 *in vitro* and *in vivo*^25^,^26^.

A potential disadvantage of the CRISPR/Cas system is the possibility of off-target effects. In the current study, we observed no obvious effects of the OVMTg2 sgRNA/Cas9 combination on potential off-target sites in the mutant chickens (Table S2). Several studies have reported frequent off-target mutagenic effects of CRISPR/Cas9 in transformed cell lines^22^,^23^; however, it has also been reported that off-target mutations are very rare and not a serious problem for CRISPR/Cas9-mediated genome editing in human stem cells^27^,^28^. Although additional targeted and/or genome-wide analyses may be required for precise evaluation of off-target effects associated with mutagenesis by CRISPR/Cas9 in chickens, our results suggest that specific gene targeting occurs with the CRISPR/Cas9 system in chicken PGCs.

Both OVA and OVM are major allergenic proteins. Of the egg white proteins, OVA is the most abundant allergenic protein, whereas OVM is the dominant allergen^29^. Hens with homozygous disruptions in these allergen genes may produce low-allergenicity eggs, which could be tolerated by a person who has egg white allergies. In particular, allergenicity of OVM has been hard to eliminate from egg whites because of the strong stability of OVM during heating and proteolytic digestion^30^,^31^; therefore, genetic ablation of *OVM* may provide a new and effective method to reduce egg white–based allergic reactions. Heterozygous mutants in *OVA* have been generated by TALEN^13^, and in the current study we established heterozygous and homozygous *OVM* mutants by CRISPR/Cas9 (Fig. 4). It will be of immediate interest to test whether eggs from the homozygous knockout hens show reduced allergenicity. Though time intensive, it is possible to envisage crossing *OVA* and *OVM* mutant chickens to produce lines carrying mutations in multiple allergy-related genes. Alternatively, given the efficient mutagenesis in chicken PGCs by CRISPR/Cas9, hens with the disruption of multiple allergenic genes may be generated more rapidly via their simultaneous disruption in PGCs.

Of the three G0 germline chimeras from this study, two (#372 and #376) were estimated to have a relatively high contribution from the mutated donor sperm, but one (#387) had a low contribution in this regard (Table 2). A difference in the proportion of genetically modified sperm in G0 individuals has frequently been observed in PGC-transplanted germline chimeras^7^,^8^ and is presumed to result from differences in donor PGC contribution in the recipient testis. The variability of germline transmission among chimeras may be attributable to variation in the number of injected PGCs, bleeding and/or vascular occlusion after PGC transplantation, and differential expansion of PGCs within the gonad^7^. Because this variability is hard to circumvent, estimation of donor contribution in G0 semen and the subsequent avoidance of chickens with low donor contribution as founders are important for the efficient establishment of genetically modified chickens in the G1 population^8^. Although donor (BPR) and recipient (WL) sperm are hard to distinguish by DNA analysis in general, we could easily estimate the relative donor contribution in G0 semen based on *OVM* sequence analysis (Table 2) because of the high frequency of *OVM* mutations in transplanted PGCs (Table 1). The evaluation of donor contribution in G0 semen without the need for test crosses can reduce the number of experimental animals needed, and thus chicken mutagenesis mediated by the CRISPR/Cas9 system in this manner may have an advantage with regard to the ethical treatment of animals.

To generate homozygous mutant chickens, the current methods of chicken genome editing (TALEN and CRISPR/Cas9) require the interbreeding of the G1 generation of heterozygous mutant male and female chickens. The interbreeding is not difficult but requires a time-consuming process that takes ~6 months for chickens of the G1 generation to reach sexual maturity. To shorten the time needed for generating homozygous mutant chickens, it is conceivable that the G0 male and female chimeras carrying mutated gametes could be interbred. In addition to males, if G0 female germline chimeras were generated, homozygous mutant chickens could be obtained in G1, one generation earlier than is achieved with the current method. Because these technologies have thus far been unsuccessful in female-derived PGCs^7^,^8^,^32^, better methods for female-derived PGCs should be developed for the rapid generation of homozygous mutant chickens. Culture and mutation methods for female-derived PGCs would also enable the introduction of mutations on the female-specific W chromosome.

We have demonstrated a simple and efficient method to generate knockout chickens using the CRISPR/Cas9 system in PGCs. Because the chicken embryo has been an important model for studying developmental biology^33^, a convenient knockout technology will be a powerful tool not only for the improvement of chicken products but also for developmental studies including chicken embryonic pattern formation, tissue morphogenesis, and organogenesis. In many types of cells and organisms, CRISPR/Cas9 has been used to generate not only knockout but also knock-in genotypes. In addition, CRISPR/Cas technology has been expanded to include various genome-engineering applications such as large-scale genomic deletions, transcriptional modification, and epigenetic regulation^34^,^35^. We envisage that many of these advances may be applicable in PGCs and chicken by modification of the method developed in our study. Expansion of the various genome-engineering technologies with the CRISPR/Cas system may be expected to lead to fundamental advances such as a greater understanding of PGC development and chicken embryonic morphogenesis, genetically based methods to improve chicken health and productivity, and production of knock-in chickens for agricultural and biomedical applications.

## Methods

### Animal experiments

All animal experiments were done strictly in accordance with the protocols approved by the institutional animal care and use committees of both National Institute of Advanced Industrial Science and Technology (AIST) and NARO Institute of Livestock and Grassland Science (NILGS). Chickens, including the White Leghorn (WL) and Barred Plymouth Rock (BPR), were maintained at the animal farm facility of NILGS.

### Plasmid construction

The plasmids expressing *hCas9* and sgRNA were generated by inserting complementary oligonucleotides corresponding to each target sequence (Fig. 1, Fig. S2, and Table S1) into the BbsI site of pX330 (Addgene, Cambridge, MA)^14^. For antibiotic selection, neomycin-, puromycin-, and zeocin-resistant genes were inserted into the NotI site of pX330. Two reporter plasmids for the SSA assay, pCAG-EGxxFP-OVA and pCAG-EGxxFP-OVM, were generated by ligating PCR-amplified genomic fragments of *OVA* (481 bp) and *OVM* (457 bp), respectively, containing the sgRNA target sites into the EcoRI site of pCAG-EGxxFP^20^. Primers for PCR amplification are shown in Table S1, and PCR was carried out with PrimeSTAR HS DNA polymerase (TaKaRa, Otsu, Japan), as follows: 98 °C for 2 min and 30 cycles of 98 °C for 10 s, 55 °C for 5 s, and 72 °C for 30 s. pCAG-EGxxFP vector and pX330/Cetn1, which was used as an experimental control for the SSA assay (see below), were kindly provided by Dr. Ikawa (Osaka University).

### SSA recombination assay

The ability of pX330 plasmids (pX330-Neo-OVATg1–4 and pX330-Neo-OVMTg1–4) to disrupt the target gene was evaluated by the SSA assay as described^20^. In brief, HEK293T cells (4 × 10^5^) were seeded into individual wells of poly-l-lysine–coated 6-well plates. After 24 h, co-transfection of pX330-sgRNA sequence–carrying plasmids and pCAG-EGxxFP target-site plasmids (0.5 μg each) was carried out with Lipofectamine 2000 (Life Technologies, Carlsbad, CA). After an additional 48 h, cells that were positive and those that were negative for EGFP fluorescence were scored in four regions (0.25 mm^2^ each), and the percentage of positive cells relative to all cells was determined.

### PGC culture and transfection

PGCs were isolated from blood of BPR embryos at stage 14–16 (Hamburger-Hamilton, HH) and incubated in forced-air incubators (P-008B Biotype; Showa Furanki, Saitama, Japan) at 38.5 °C and 60–80% relative humidity. Cells were cultured on Mitomycin C–treated buffalo rat liver (BRL) feeder cells in BRL-conditioned medium with supplements as described^36^. PGC transfection for genome editing was carried out using Lipofectamine 2000 according to the manufacturer’s protocol with these minor modifications: (i) 1.6 μg of pX330-sgRNA sequence–carrying plasmid, and (ii) Lipofectamine 2000 reagents were diluted in 100 μl of OPTI-MEM (Thermo Fisher Scientific, Waltham, MA) and then were incubated with 0.5–1 × 10^5^ PGCs for 5 min, followed by a 2-h incubation at 37 °C with 400 μl of antibiotic-free BRL-conditioned medium. Then, PGCs were transferred to 6-well plates with BRL feeder cells and were cultured at 37 °C, 5% CO_2_. PGCs were treated with or without 500 μg/ml neomycin (Nacalai, Kyoto, Japan), 1 μg/ml puromycin (InvivoGen, San Diego, CA), or 50 μg/ml zeocin (InvivoGen) from day 2 to day 4 after transfection to enrich for pX330-transfected cells. The remaining PGCs were then transferred to antibiotic-free medium and allowed to proliferate for transplantation and analysis.

### Genomic DNA sequence analysis

Genomic DNA was extracted from PGCs, chicken semen, or shafts of chick feathers using the DNeasy Blood and Tissue Kit (Qiagen, Valencia, CA). Genomic fragments containing sgRNA target sites were PCR amplified using MightyAmp DNA polymerase Ver.2 (TaKaRa) with specific primer sets (Table S1), TA cloned into pGEM-T Easy vector (Promega, Madison, WI), and sequenced with the ABI 310 Genetic Analyzer (Applied Biosystems, Foster City, CA). PCR conditions were as follows: 98 °C for 2 min and 40 cycles of 98 °C for 10 s, 60 °C for 10 s, and 72 °C for 30 s. For screening of *OVM* mutant chickens (G1), PCR products were purified using QIAquick PCR purification kit (Qiagen) and sequenced with specific primers (Table S1).

### Gamma irradiation, PGC microinjection, and embryo culture

WL eggs were irradiated at 1 Gy/min with ^137^Cs with a Gammacell 40 irradiator (Atomic Energy of Canada Ltd., Chalk River, ON, Canada). Irradiated embryos were allowed to develop to stage 13–15 HH, and then 1,000–2,000 PGCs, the majority of which contained *OVM* mutations based on an evaluation of mutation frequency (see Fig. 4a), were injected into the bloodstream of embryos as described^36^. The male embryos, as detected by PCR^32^, were allowed to develop in forced-air incubators with tilting at a 60° angle two times each hour at 38.5 °C and 60–80% relative humidity until hatch-out.

### Progeny test

Male putative chimeric chickens were raised to sexual maturity. The proportion of mutated sperm was estimated by sequence analysis of the *OVM* target site region in TA-cloned PCR amplicons from semen genomic DNA. Two roosters estimated to have >90% mutated cells in their semen were crossed with wild-type female BPR (i/i) chickens by artificial insemination. Offspring that were derived from donor (BPR; i/i) and recipient (WL; I/I) PGCs were identified by their black (i/i) and white (I/i) feather color, respectively. The sequences of *OVM* target site regions in donor-derived offspring were analyzed as described above. For G2 production, a sexually mature *OVM*^+/−^ male and female were crossed.

### Off-target analysis

Potential off-targets of OVMTg2 sgRNA were predicted by a BLAST search of the 13 nucleotides located 5′ to the PAM motif consisting of 5′-NGG-3′^37^ or of the 19 nucleotides 5′ to a possible PAM motif, 5′-NAG-3′^38^. The search was performed against the chicken genome sequence (Gallus_gallus-4.0 assembly; GenBank Assembly ID GCA_000002315.2) in the Ensembl database (http://www.ensembl.org/index.html), and three potential off-target sites were identified with the highest homology score in each criterion (Table S2). Genomic DNA from 19 mutant chickens was analyzed for off-target effects by PCR amplification, followed by sequence analysis (Table S1).

### Fragment analysis

Genomic DNA from G1 parents and G2 offspring was PCR amplified with 6-FAM 5′-labeled reverse primer and unlabeled forward primer against *OVM* (Table S1). PCR conditions were as follows: 98 °C for 2 min and 35 cycles of 98 °C for 10 s and 68 °C for 20 s. PCR products and HEX-labeled control *OVM* amplicon from the wild-type genome were analyzed using genescan fragment analysis at Macrogen Japan (Kyoto, Japan). We used Peak Scanner Software V2.0 (Applied Biosystems) to analyze the results.

## Additional Information

**How to cite this article**: Oishi, I. *et al.* Targeted mutagenesis in chicken using CRISPR/Cas9 system. *Sci. Rep.* **6**, 23980; doi: 10.1038/srep23980 (2016).

## Supplementary Material

Supplementary Information

## Figures and Tables

**Table 1:** Efficiency of induction of *OVA* and *OVM* mutations in PGCs.

Vector	Antibiotic	Analyzed clones	Mutated clones	Mutation frequency (%)
OVATg3-neo	–	45	3	6.7
OVATg3-neo	neomycin (0.5 mg/ml)	29	10	34
OVATg3-puro	puromycin (1 μg/ml)	12	12	100
OVATg3-zeo	zeocin (50 μg/ml)	12	11	92
OVMTg2-neo	–	24	0	0
OVMTg2-neo	neomycin (0.5 mg/ml)	24	3	13
OVMTg2-puro	puromycin (1 μg/ml)	23	21	91
OVMTg2-zeo	zeocin (50 μg/ml)	12	11	92

**Table 2:** Efficiency of *OVM* mutation induction in G0 semen and G1 offspring.

Parent (G0)	Number of mutated TA clones from G0 semen (%)^a^	Number of donor-derived chicks (G1) (%)^b^	Mutants from donor-derived chicks (%)
#372	12/13 (92%)	33/42 (79%)	19/33 (58%)
#376	15/16 (94%)	29/43 (67%)	14/29 (48%)
#387^c^	3/20 (15%)*	NT	NT

^a^The OVMTg2 region was amplified from rooster semen, and individual clones were sequence analyzed. Values represent the percentage of mutated sequences relative to the total number of clones analyzed.

^b^The percentage of donor-derived chicks (i.e., black-feathered chicks) was determined relative to the total number of chicks.

^c^NT, not tested.

^*^Significantly different compared with #372 and #376, as calculated by a chi-square test (P < 0.001).
