# Supplementary material for: Targeted mutagenesis in chicken using CRISPR/Cas9 system
Source: Sci Rep. 2016 Apr 6;6:23980. doi: 10.1038/srep23980 (PMC4822141; doi:10.1038/srep23980)
Supplement: Supplementary Information [file srep23980-s1.pdf]

Supplementary Information

Targeted mutagenesis in chicken using CRISPR/Cas9 system

Isao Oishi[1]*, Kyoko Yoshii[1], Daichi Miyahara[2], Hiroshi Kagami[2], Takahiro Tagami[3]*

[1]Biomedical Research Institute, National Institute of Advanced Industrial Science and Technology, 1-8-31, Midorioka, Ikeda, Osaka 563-8577, Japan

[2]Faculty of Agriculture, Shinshu University, 8304 Minamiminowa, Nagano 399-4598, Japan,

[3]Animal Breeding and Reproduction Research Division, National Agriculture and Food Research Organization, Institute of Livestock and Grassland Science, 2 Ikenodai, Tsukuba, Ibaraki 305-0901, Japan

* Authors for correspondence oishi-i@aist.go.jp, tagami@affrc.go.jp

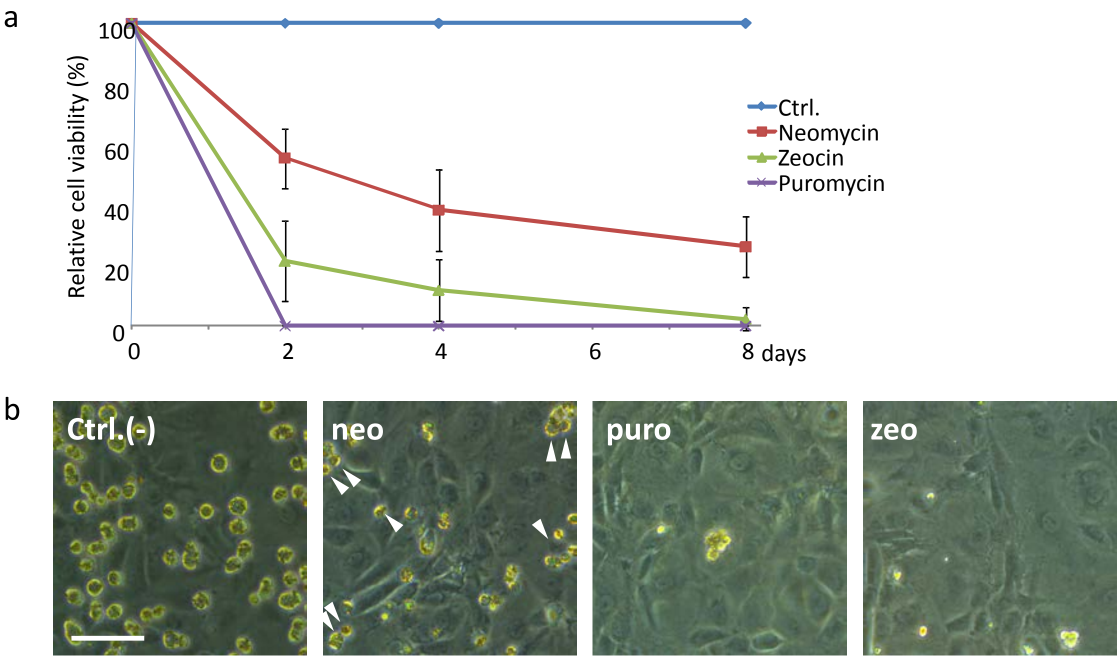

Figure S1. Toxic effect of neomycin, puromycin, and zeocin on untransfected PGCs. (a) Relative viability of PGCs treated with antibiotics. PGCs were seeded at a density of 2 × 10^4 cells/well in a 12-well plate and cultured with or without (ctrl.) antibiotics (0.5 mg/ml neomycin, 1 μg/ml puromycin, or 50 μg/ml zeocin) for 2 days, and then were washed and cultured without antibiotics for an additional 6 days. PGCs were stained with trypan blue (0.4% w/v in PBS) on the indicated days in culture, and the number of unstained (i.e., living) cells was scored. Viability relative to control PGCs is plotted. Each point represents the mean ± SD (bar) from three wells. (b) Phase contrast image of PGCs after 8 days in culture as in (a). Neomycin-treated PGCs include round-shaped living cells (arrowheads). Scale bar = 100 μm.

a

```
                                 CVHTg2
gtccaaaggtgacttatgtcccccctcctccacctgaagatgaacagtccatctttgcat
   P  K  V  T  Y  V  P  P  P  P  P  E  D  E  Q  S  I  F  A

                                         CVHTg1
gttatcagtcaggaattaattttgacaagtatgatgaatgtgctgttgagatgtcaggac
C  Y  Q  S  G  I  N  F  D  K  Y  D  E  C  A  V  E  M  S  G
```

b

```
CVHTg1
wt:     gacaagtatgatGAATGTGCTGTTGAGATGTCaggacttgaccctccagcaccatt
mt -1   gacaagtatgatgaatgtgctgttgag-tgtcaggacttgaccctccagcaccatt x3
mt -1   gacaagtatgatgaatgtgctgttgaga-gtcaggacttgaccctccagcaccatt x1
mt -2   gacaagtatgatgaatgtgctgttga--tgtcaggacttgaccctccagcaccatt x3
mt -3   gacaagtatgatgaatgtgctgttg---tgtcaggacttgaccctccagcaccatt x1
mt -3   gacaagtatgatgaatgtgctgttga---gtcaggacttgaccctccagcaccatt x1
mt -4   gacaagtatgatgaatgtgctgttg----gtcaggacttgaccctccagcaccatt x2
mt -8   gacaagtatgatgaatgtgctgttgagat--------ttgaccctccagcaccatt x2
mt -12  gacaagtatgatgaatgtgctgttgagat------------ccctccagcaccatt x1
mt -17  gacaagtatgatgaatgtgct-----------------tgaccctccagcaccatt x2
mt -22  gacaagtatgatgaatgtgctgttgag----------------------caccatt x1

CVHTg2
wt:     tcccccctcctCCACCTGAAGATGAACAGTCCatctttgcatgttatcagtcagga
mt -1   tcccccctcctcc-cctgaagatgaacagtccatctttgcatgttatcagtcagga x2
mt -1   tcccccctcctcca-ctgaagatgaacagtccatctttgcatgttatcagtcagga x1
mt -2   tcccccctcctc--cctgaagatgaacagtccatctttgcatgttatcagtcagga x2
mt -4   tcccccctcctcca----aagatgaacagtccatctttgcatgttatcagtcagga x1
mt -8   tcccccctcctcc--------atgaacagtccatctttgcatgttatcagtcagga x2
mt -9   tcccc---------cctgaagatgaacagtccatctttgcatgttatcagtcagga x1
```

c

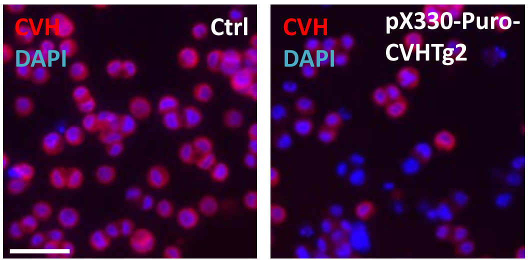

Figure S2. CRISPR/Cas9-mediated mutation of chicken vasa homolog (CVH) in PGCs. (a) Schematic representation of sgRNAs targeting CVH exon 8. DNA and amino acid sequences are shown in lowercase and uppercase letters, respectively. The two sgRNA targeting sites (CVHTg1 and CVHTg2) are represented by black bars above the nucleotide sequence. Adjoining protospacer adjacent motif (PAM) sequences are in red. (b) Sequence analysis of CVHTg1 and CVHTg2-induced deletion mutations in PGCs. Cultured PGCs were transiently transfected with pX330-puro-CVHTg1 or -CVHTg2, selected with 1 μg/ml puromycin at 2–4 days post-transfection, and then cultured without puromycin. Part of the PGCs were collected, and *CVH* sequence around target sites were PCR amplified and analyzed following TA cloning. We found mutations around *CVH* Tg1 and Tg2 sites with frequencies of 89% (n = 19) and 90% (n = 10), respectively**.** The wild-type CVH sequence (wt) is shown at the top of each panel. The sgRNA-targeted locus is indicated in capital letters in red; the PAM sequence is in green. The number of deleted nucleotides (–1 to –22) is indicated to the left of each sequence. Deleted nucleotides are shown by dashes. The number of identical mutant clones is shown to the right of each sequence. (c) Immunostaining of PGCs for CVH (red) with DAPI (blue). The majority of PGCs transfected with pX330-Puro-CVHTg2 followed by transient puromycin selection (right panel) had reduced or absent CVH expression. The Immunostaining was carried out as described previously[31]. Scale bar = 100 μm.

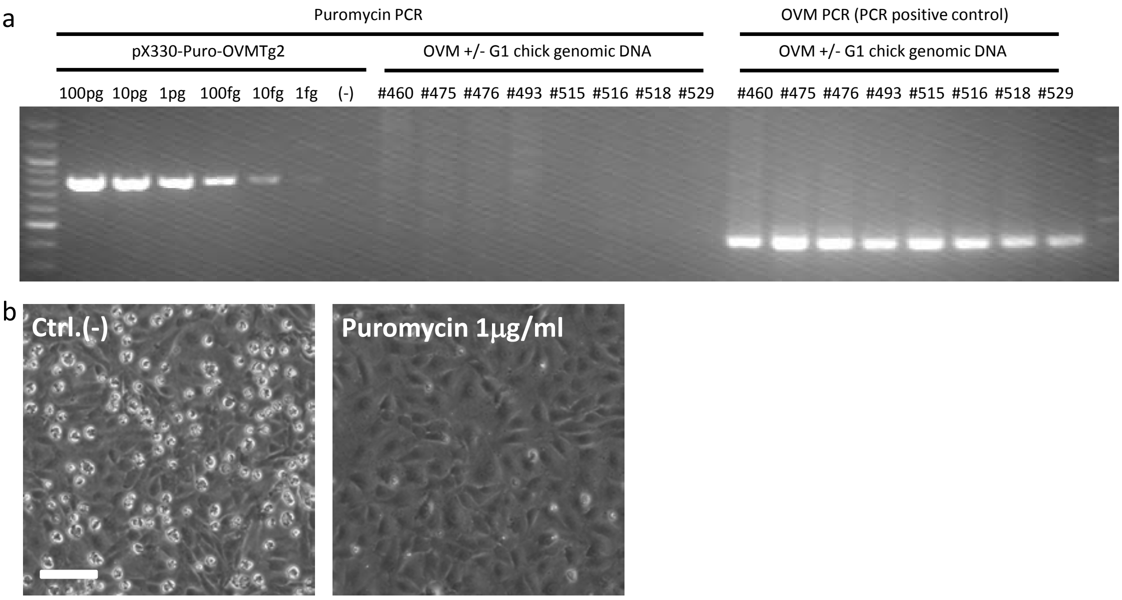

Figure S3. Detection of transfected plasmid (pX330-Puro-OVMTg2) in G1 mutant chicks and untlansplanted PGCs. (a) Genomic PCR of G1 mutant chicks. The primer set for amplification of the puromycin resistance gene (see Table S1) could detect 10 fg of pX330-Puro-OVM2 vector. The puromycin resistance gene could not be detected in 10 ng of genomic DNA from eight *OVM*$^{+/-}$ G1 chicks (#460-#529). Endogenous *OVM* was detected under the same PCR conditions (98°C for 2 min and 35 cycles of 98°C for 10 s, 60°C for 10 s, and 72°C for 30 s) with OVMTg2 target site PCR primers (Table S1). (b) Puromycin sensitivity of the transplantation-competent PGCs. The remaining PGCs (i.e., those that were not transplanted but were stored in liquid nitrogen) were allowed to proliferate for 5 days and then were cultured with or without 1 μg/ml puromycin for 1 week. Phase contrast images show that round-shaped (i.e., living) PGCs are rarely seen in the presence of puromycin (right). Scale bar = 100 μm.

Table S1 Primers used in this study

Ovalbumin	Forward[a]	Reverse[a]
pX330-sgRNA/OVATg1[b]	caccGACAACTCAGAGTTCACCAT	aaacATGGTGAACTCTGAGTTGTC
pX330-sgRNA/OVATg2[b]	caccGAGTTCACCATGGGCTCCAT	aaacATGGAGCCCATGGTGAACTC
pX330-sgRNA/OVATg3[b]	caccGCTCTAGCCATGGTATACCT	aaacAGGTATACCATGGCTAGAGC
pX330-sgRNA/OVATg4[b]	caccgATGGCTAGAGCTGACATGA	aaacTCATGTCAGCTCTAGCCATC
pCAG-EGxxFP/OVA[c]	ttatgaaGAATTCttgaacatgcacatgagaggtg	tggtttcaGGAATTCagcctctgagctatgcagtt
OVATg3 target site PCR amplification	ctatgtacagcattccatccttac	ttcagcctctgagctatgcagtt
OVATg3 target site sequencing	aactcagagttcaccatgggc	tttccaagggacattatcatac
Ovomucoid	Forward[a]	Reverse[a]
pX330-sgRNA/OVMTg1[b]	caccGTTGTCAGGTGGACTGCAGT	aaacACTGCAGTCCACCTGACAAC
pX330-sgRNA/OVMTg2[b]	caccGTTTCCCAACGCTACAGACA	aaacTGTCTGTAGCGTTGGGAAAC
pX330-sgRNA/OVMTg3[b]	caccGACTCCATCGGTACCACAGA	aaacTCTGTGGTACCGATGGAGTC
pX330-sgRNA/OVMTg4[b]	caccGTTGGTGTAAGTGACTCCAT	aaacATGGAGTCACTTACACCAAC
pCAG-EGxxFP/OVM[c]	tgatagatGAATTctggtttatcacatggggacct	acagtaaGAATTCtgatgtctaggcaaccgagtgt
OVMTg2 target site PCR amplification	ctggtttatcacatggggacct	tgatgtctaggcaaccgagtgt
OVMTg2 target site sequencing	ctacaaaatgtcactttgtcc	gcagtacacacatgctgtag
OVM off-target 1	ccctattacagtgcaatcagtctggctgag	cttttgttacctttacagctctgtgtctgac
OVM off-target 2	gtaaaacaaggtaaggcctgcttgctctcg	tgccttttcatcatagtaggaactctgagat
OVM off-target 3	aactttggactgccgttatccttctctcag	gccaaccactaaatcaggcacttgatcagg
OVM off-target 1sequencing	tgaagctctgatatgcttagg	
OVM off-target 2 sequencing	atttagaacagccaatgtggaag	
OVM off-target 3 sequencing	actcccaaaaaccatgctagg	
OVM fragment analysis[d]	ttctttgtcaggtggactgcagtag	ctgcagtacacacatgctgtag
puromycin PCR	gcgccaccttctactcctcccctagtc	agacccttgccctggtg
Chicken Vasa homolog	Forward[a]	Reverse[a]
pX330-sgRNA/CVHTg1[b]	caccGAATGTGCTGTTGAGATGTC	aaacGACATCTCAACAGCACATTC
pX330-sgRNA/CVHTg2[b]	caccGACTGTTCATCTTCAGGTGG	aaacCCACCTGAAGATGAACAGTC
CVHTg2 target site PCR amplification	ttcctgagcaaatgtgagaatgcg	tggaagcaaaattacatactac
CVHTg2 target site sequencing	aaggaaaatactattgacttc	atcacagaatcacacttaatac

[a]All primers are written 5' to 3'. [b] Ttarget sgRNA are in uppercase.

[c] EcoRI site for pCAG-EGxxFP plasmid construction are in uppercase.

[d] 5' end of Rverse primer was labbeled with FAM or HEX

Table S2 Sequence analysis of off-target regions after OVMTg2 CRISPR/Cas9

	Chr	Locus	Seed sequence[a]	PAM	Frequency of indels in PGC[b]	Frequency of indels in G1[c]
OVMTg2	13	9,896,628	TTTCCCAACGCTACAGACA	AGG		
OVM off-target 1	1	114,927,708	CTGCAGCACGCTACAGACA	TGG	0/24	0/19
OVM off-target 2	7	12,053,381	ATAAATTACGCTACAGACA	AGG	0/24	0/15
OVM off-target 3	4	46,095,298	TGGCCCAACGCTACAGACA	CAG	0/24	0/9

[a]Identical nucleotides in putative off-target sequences are underlined.

[b]Off-target regions were amplified from genomic DNA of untransplanted PGCs, TA cloned, and individual clones were sequence analyzed. Number of indel clones relative to the number of analyzed TA clones are represented.

[c]Number of indels relative to the number of analyzed sequences.
